# Cerebellar Stellate Cell Excitability Is Coordinated by Shifts in the Gating Behavior of Voltage-Gated Na^+^ and A-Type K^+^ Channels

**DOI:** 10.1523/ENEURO.0126-19.2019

**Published:** 2019-06-04

**Authors:** Ryan P.D. Alexander, John Mitry, Vasu Sareen, Anmar Khadra, Derek Bowie

**Affiliations:** 1 Integrated Program in Neuroscience, McGill University, Montréal, Quebec H3A 2B4, Canada; 2 Department of Pharmacology and Therapeutics, McGill University, Montréal, Quebec H3G 1Y6, Canada; 3 Department of Physiology, McGill University, Montréal, Quebec H3G 1Y6, Canada

**Keywords:** A-type potassium channel, action potential, cerebellum, computational modeling, sodium channel, stellate cell

## Abstract

Neuronal excitability in the vertebrate brain is governed by the coordinated activity of both ligand- and voltage-gated ion channels. In the cerebellum, spontaneous action potential (AP) firing of inhibitory stellate cells (SCs) is variable, typically operating within the 5- to 30-Hz frequency range. AP frequency is shaped by the activity of somatodendritic A-type K^+^ channels and the inhibitory effect of GABAergic transmission. An added complication, however, is that whole-cell recording from SCs induces a time-dependent and sustained increase in membrane excitability making it difficult to define the full range of firing rates. Here, we show that whole-cell recording in cerebellar SCs of both male and female mice augments firing rates by reducing the membrane potential at which APs are initiated. AP threshold is lowered due to a hyperpolarizing shift in the gating behavior of voltage-gated Na^+^ channels. Whole-cell recording also elicits a hyperpolarizing shift in the gating behavior of A-type K^+^ channels which contributes to increased firing rates. Hodgkin–Huxley modeling and pharmacological experiments reveal that gating shifts in A-type K^+^ channel activity do not impact AP threshold, but rather promote channel inactivation which removes restraint on the upper limit of firing rates. Taken together, our work reveals an unappreciated impact of voltage-gated Na^+^ channels that work in coordination with A-type K^+^ channels to regulate the firing frequency of cerebellar SCs.

## Significance Statement

The cerebellum is a brain region that fulfills critical roles in motor function in adults as well as being linked to neurodevelopmental disorders in the developing brain. Significant attention has been directed toward understanding connectivity within the cerebellum and how its neuronal circuits are regulated. Stellate cells (SCs) are inhibitory GABAergic interneurons that make-up neuronal circuits that control the output from the cerebellar cortex by regulating the firing properties of Purkinje cells. The strength of GABAergic inhibition of Purkinje cells is governed by the excitability of SCs which fire action potentials (APs) at a wide range of frequencies. Our study reveals an unappreciated role of voltage-gated sodium channels that work in coordination with A-type K^+^-channels to establish SC firing rates.

## Introduction

Cerebellar stellate cells (SCs) are GABAergic interneurons that exert inhibitory tone onto both Purkinje cells (PCs) and SCs to shape motor function in awake, behaving animals (Astorga et al., 2017; Gaffield and Christie, 2017). Investigations into the physiologic activity of SCs both *in vitro* and *in vivo* have estimated their action potential (AP) firing rates to be in the range of 5–30 Hz (Armstrong and Rawson, 1979; Midtgaard, 1992; Häusser and Clark, 1997; Carter and Regehr, 2002), with some studies reporting even lower spontaneous rates (Jörntell and Ekerot, 2003; Liu et al., 2014). SCs are also highly sensitive to minimal amounts of synaptic input (Carter and Regehr, 2002; Jörntell and Ekerot, 2003), suggesting that the excitability of SCs is finely tuned to ensure that their target cells receive robust and reliable feedforward inhibition.

Several molecular mechanisms have been shown to modulate the excitability of SCs. For example, elevations in cytosolic Ca^2+^ mediated by T-type voltage gated Ca^2+^ channels (VGCCs) have been shown to dynamically regulate the firing rates of SCs by modulation of somatodendritic A-type K^+^ channels (Molineux et al., 2005; Anderson et al., 2013). Firing rates are further controlled by neurochemical transmission. The inhibitory tone of GABA_A_ receptors constrains AP firing (Häusser and Clark, 1997) whereas the prolonged depolarization by NMDA-type ionotropic glutamate receptors (Liu et al., 2014) activates axonal VGCCs (Christie and Jahr, 2008) and promotes GABA release (Glitsch and Marty, 1999; Duguid and Smart, 2004; Liu and Lachamp, 2006). To complicate matters, the firing rates of SCs are also affected by patch-clamp recording conditions. The intrinsic excitability of SCs increases in a time-dependent manner in cell-attached recordings (Alcami et al., 2012). The molecular events that give rise to this increase in excitability are still not fully understood, although observations in other cell types have demonstrated that patch breakthrough during whole-cell recording can cause unintended changes to ion channel gating and activity, including an effect on voltage-gated Na^+^ channels (Fenwick et al., 1982; Dani et al., 1983; Fernandez et al., 1984; Vandenberg and Horn, 1984; Townsend et al., 1997). Whether a similar mechanism accounts for increased firing in SCs in whole-cell recordings has yet to be investigated.

Here, we have elucidated the molecular events responsible for the increase in excitability of SCs in whole-cell recording conditions. Using a combination of brain slice patch-clamp electrophysiology and Hodgkin–Huxley modeling, we show that shifts in the gating properties of voltage-gated Na^+^ channels cause an increase in SC excitability by promoting AP firing at more hyperpolarized potentials. These events occur concurrently with a hyperpolarizing shift in A-type K^+^ channel gating which reduces the number of channels available for activation, and thus contributes to increased AP firing. Taken together, our data identify an unappreciated role of voltage-gated Na^+^ channels that work in coordination with somatodendritic A-type K^+^ channels to upregulate SC excitability on whole-cell recording.

## Materials and Methods

### Ethical approval

All experiments have been approved by the Animal Care Committee of McGill University and were performed in accordance with the guidelines of the Canadian Council on Animal Care.

### Animals

Wild-type mice with a C57BL/6J background (RRID: IMSR_JAX:000664) were obtained from The Jackson Laboratory and maintained as a breeding colony at McGill University. Both male and female wild-type mice were used for experiments and ranged from postnatal day 18 to 30.

### Slice preparation

Mice were anesthetized with isoflurane and immediately decapitated. A block of cerebellar vermis was rapidly dissected from the mouse head and submerged in ice-cold cutting solution perfused with carbogen gas (95% O_2_, 5% CO_2_). Cutting solution contains: 235 mM sucrose, 2.5 mM KCl, 1.25 mM NaH_2_PO_4_, 28 mM NaHCO_3_, 0.5 mM CaCl_2_, 7 mM MgCl_2_, 28 mM D-glucose, 1 mM ascorbic acid, and 3 mM sodium pyruvate (pH 7.4; 305–315 mOsmol/l). The block of vermis is then fastened to a platform, transferred to the slicing chamber and again submerged in ice-cold cutting solution, bubbled with carbogen throughout the remainder of the procedure. Thin slices of cerebellar vermis (300 µm) were obtained with a vibrating tissue sectioner (Leica VT1200; Leica Instruments). The slices were transferred to oxygenated artificial CSF (aCSF) and held at room temperature (21°C–23°C) for at least 1 h before recordings were performed. aCSF contained the following: 125 mM NaCl, 2.5 mM KCl, 1.25 mM NaH_2_PO_4_, 26 mM NaHCO_3_, 2 mM CaCl_2_, 1 mM MgCl_2_, and 25 mM D-glucose (pH 7.4; 305–315 mOsmol/l).

### Electrophysiology

Slice experiments were performed on an Olympus BX51 upright microscope (Olympus) equipped with differential interference contrast/infrared optics. Whole-cell patch-clamp recordings were made from visually-identified stellate, granule or Purkinje cells in acute sagittal slices of cerebellar vermis. SCs were distinguished from misplaced or migrating granule, glial or basket cells by their small soma diameter (8–9 µm), location in the outer two-thirds of the molecular layer and whole-cell capacitance measurement (4–12 pF). Granule cells (GCs) were identified based on location in the granule layer (immediately less superficial to the Purkinje layer), small soma size and whole-cell capacitance measurement (1–3 pF). Purkinje cells were identified based on large relative size, location in the Purkinje monolayer and whole-cell capacitance measurement (25–35 pF). Patch pipettes were prepared from thick-walled borosilicate glass (GC150F-10, OD 1.5 mm, ID 0.86 mm; Harvard Apparatus Ltd) and had open tip resistances of 4–7 MΩ when filled with an intracellular recording solution. Recordings were performed using a Multiclamp 700A amplifier (Molecular Devices) at a holding potential of –70 mV (stellate and Purkinje) or –80 mV (granule). Series resistance and whole-cell capacitance were estimated by cancelling the fast transients evoked at the onset and offset of brief (10 ms) 5-mV voltage-command steps. Access resistance during whole-cell recording (7–25 MΩ) was compensated between 60 and 80% and checked for stability throughout the experiments (∼15% tolerance). Recordings where pipette offset changed by >3 mV were excluded. Liquid junction potential was not corrected for. The bath was continuously perfused at room temperature (21°C–23°C) with aCSF at a rate of 1–2 mL/min. Currents were filtered at 5 kHz with an eight-pole low-pass Bessel filter (Frequency Devices) and digitized at either 25 or 100 kHz with a Digidata 1322A data acquisition board and Clampex 10.1 (pClamp, RRID:SCR_011323) software. For voltage-clamp recordings, the online P/N leak-subtraction suite in Clampex 10.1 was used to assess voltage-gated responses.

For recording voltage dependence of activation of A-type K^+^ current ($I_{A}$) a protocol was applied consisting of 500-ms steps evoked from a holding potential of –100 mV, ranging from –100 to +20 mV in 10-mV increments. An inactivation protocol was applied consisting of 500-ms prepulse steps ranging from –120 to –30 mV in 5-mV increments, followed by a probe step to –20 mV to assess channel availability. For recording voltage dependence of activation of delayed rectifier K^+^ current ($I_{K}$), a protocol was applied consisting of 1-s steps evoked from a holding potential of –50 mV, ranging from –50 to +40 mV in 5-mV increments. The holding potential of –50 mV was chosen to selectively inactivate $I_{A}$, because of its relatively hyperpolarized V_1/2_ inactivation, and study a more isolated $I_{K}$. For recording voltage dependence of activation of Na^+^ current ($I_{Na}$), a protocol designed to circumvent space-clamp errors in neurons was used (Milescu et al., 2010) consisting of a 5-ms suprathreshold step from –80 to –35 mV to evoke an action current, followed by a ∼1-ms step to –60 mV, followed by 100-ms steps ranging from –80 to +30 mV in 5-mV increments. To measure steady-state channel availability, an inactivation protocol consisting of 100-ms steps ranging from –110 to –20 mV in 5-mV increments, followed by a probe step to –20 mV.

### Recording solutions

All chemicals were obtained from Sigma Aldrich unless otherwise indicated. Internal pipette solution for most current-clamp experiments as well as voltage-clamp experiments, both whole-cell and nucleated patch, examining $I_{K}$ in SCs contained: 126 mM K-gluconate, 5 mM HEPES, 4 mM NaCl, 15 mM D-glucose, 0.05 mM CaCl_2_, 1 mM MgSO_4_, 0.15 mM K_4_-BAPTA, 2 mM Mg-ATP, and 0.1 mM Na-GTP (adjusted to pH 7.4 with KOH, 300–310 mOsmol/l). $I_{K}$ experiments had external aCSF supplemented with 100 nM tetrodotoxin (TTX) to block AP firing, as well as 5 mM 4-aminopyridine (4-AP) to limit the activity of $I_{A}$-mediating channels. In one set of experiments, all K-gluconate in this internal solution was substituted for K-methanesulfonate to examine the effect of different anions on intrinsic membrane properties. Pipette solution for voltage-clamp experiments, both whole-cell and nucleated patch, examining $I_{A}$ contained: 140 mM KCl, 10 mM HEPES, 2.5 mM MgCl_2_, and 0.15 mM K_4_-BAPTA (adjusted to pH 7.4 with KOH, 300–310 mOsmol/l). For these experiments, the external aCSF was supplemented with 5 mM TEA-Cl and 2 mM CsCl to block non-$I_{A}$-mediating K^+^ channels, and 100 nM TTX to block AP firing. Pipette solution for voltage-clamp experiments examining $I_{Na}$ contained: 110 mM Cs-methanesulfonate, 5 mM HEPES, 4 mM NaCl, 15 mM D-glucose, 0.05 mM CaCl_2_, 0.15 mM Cs_4_-BAPTA, 4 mM Mg-ATP, 0.1 mM Na-GTP, 10 mM TEA-Cl, and 10 mM 4-AP (adjusted to pH 7.4 with CsOH, 300–310 mOsmol/l). Pipette solution for voltage-clamp experiments examining $I_{Na}$ in excised membrane patches contained: 140 mM CsCl, 10 mM HEPES, 10 mM EGTA, 2 mM MgCl_2_, and 2 mM Na_2_-ATP (adjusted to pH 7.4 with CsOH, 300–310 mOsmol/l). Free Ca^2+^ concentration for all current-clamp internal solutions was calculated to be ∼100 nM using MaxChelator freeware (Bers et al., 2010). For all experiments investigating $I_{Na}$, the external aCSF was supplemented with 100 µM CdCl_2_ and 1 µM Mibefradil dihydrochloride (Tocris Bioscience) to block voltage-gated calcium channels. Additional current-clamp experiments were performed in the presence of hyperpolarization-activated cyclic nucleotide-gated channel (HCN) blocker ZD 7288 in the aCSF (20 µM; Tocris Bioscience). Except where indicated, all experiments were performed in the presence of fast excitatory and inhibitory synaptic blockers: NMDA receptor antagonist D-(-)-2-amino-5 phosphonopentanoic acid (D-APV; 10 µM), AMPA/kainate receptor antagonist 2,3-dioxo-6-nitro-1,2,3,4-tetrahydrobenzo[f]quinoxaline-7-sulfonamide (NBQX; 10 µM), and GABA_A_ receptor antagonist bicuculline (BIC; 10 µM), all of which were purchased from Abcam.

### Mathematical model

A modified Hodgkin–Huxley type model was adopted from Molineux et al. (2005) and Anderson et al. (2010). The model consists of five ionic currents, including $I_{Na}$, $I_{K}$, $I_{A}$, T-type Ca^2+^ current ($I_{T}$) and nonspecific leak current ($I_{L}$). The A-type K^+^ and T-type Ca^2+^ current were added in Molineux et al. (2005) to capture biphasic first spike latency profile. The resulting voltage equation associated with this model is expressed as

$$C_{m}\frac{dV}{dt}=-\left[I_{Na}+I_{K}+I_{A}+I_{T}+I_{L}\right]+I_{app},$$

where $C_{m}$ is the membrane capacitance per unit area and $I_{app}$ is the applied current. The kinetics of the various ionic currents included in the model are as described below (Molineux et al., 2005; Anderson et al., 2010).

(1) Fast activating Na^+^ current:

$$I_{Na}={\bar{g}}_{Na}m_{\infty }^{3}h\left(V-V_{Na}\right),$$

with maximum conductance ${\bar{g}}_{Na}$ and Nernst potential for Na^+^ $V_{Na}$. Its gating is governed by both the steady state activation function

$$m_{\infty }={\left(1+e^{-(V-v_{m})/s_{m}}\right)}^{-1}$$

and the inactivation variable h satisfying the dynamic equation

$$\frac{dh}{dt}=\frac{h_{\infty }(V)-h}{\tau_{h}(V)},$$

where $h_{\infty }\left(V\right)$ is the steady state inactivation function given by

$$h_{\infty }={\left(1+e^{(V-v_{h})/s_{h}}\right)}^{-1}$$

and $\tau_{h}\left(V\right)$ is its time constant described by the Lorentzian function

$$\tau_{h}\left(V\right)=y_{0}+\frac{2Aw}{4\pi {\left(V-V_{c}\right)}^{2}+w^{2}}.$$

(2) Delayed rectifier K^+^ current:

$$I_{K}={\bar{g}}_{K}n^{4}(V-V_{K}),$$

with maximum conductance ${\bar{g}}_{K}$ and Nernst potential for K^+^ $V_{K}$. The gating of this current is governed by the activation variable n only, which satisfies the dynamic equation

$$\frac{dn}{dt}=\frac{n_{\infty }(V)-n}{\tau_{n}(V)},$$

where $n_{\infty }\left(V\right)$ is the steady state activation function given by

$$n_{\infty }={\left(1+e^{-\left(V-v_{n}\right)/s_{n}}\right)}^{-1}$$

and $\tau_{n}\left(V\right)$ is its time constant defined by

$$\tau_{n}\left(V\right)=\frac{6}{1+e^{(V+23)/15}}.$$

(3) A-type K^+^ current:

$$I_{A}={\bar{g}}_{A}n_{A}h_{A}\left(V-V_{K}\right)$$

with maximum conductance ${\bar{g}}_{A}$ and Nernst potential $V_{K}$. Its activation/inactivation kinetics are governed by the gating variables $n_{A}$ and $h_{A}$, respectively, each satisfying the dynamic equations

$$\frac{dn_{A}}{dt}=\frac{n_{A,\infty }\left(V\right)-n_{A}}{\tau_{n_{A}}}$$

and

$$\frac{dh_{A}}{dt}=\frac{h_{A,\infty }\left(V\right)-h_{A}}{\tau_{h_{A}}}$$

where $n_{A,\infty }$ and $h_{A,\infty }$ are, respectively, the steady state activation and inactivation functions given by

(1) $$n_{A,\infty }={\left(1+e^{-\left(V-v_{n_{A}}\right)/s_{n_{A}}}\right)}^{-1}\text{and}h_{A,\infty }={\left(1+e^{\left(V-v_{h_{A}}\right)/s_{h_{A}}}\right)}^{-1}$$

and $\tau_{n_{A}}$ and $\tau_{h_{A}}$ are their corresponding time constants that are independent of membrane voltage V.

(4) Fast activating T-type Ca^2+^ current:

$$I_{T}={\bar{g}}_{T}m_{T,\infty }h_{T}\left(V-V_{Ca}\right),$$

with maximum conductance ${\bar{g}}_{T}$ and Nernst potential for Ca^2+^ $V_{Ca}$. Its gating is governed by the steady state activation function

$$m_{T,\infty }={\left(1+e^{-(V-v_{m_{T}})/s_{m_{T}}}\right)}^{-1}$$

and the inactivation variable $h_{T}$ satisfying the dynamic equation

$$\frac{dh_{T}}{dt}=\frac{h_{T,\infty }(V)-h_{T}}{\tau_{h_{T}}},$$

where $h_{\infty }\left(V\right)$ is the steady state inactivation function given by

$$h_{T,\infty }={\left(1+e^{(V-v_{h_{T}})/s_{h_{T}}}\right)}^{-1}$$

and $\tau_{h_{T}}$ is its voltage-independent time constant.

(5) Non-specific leak current:

$$I_{A}={\bar{g}}_{L}\left(V-V_{L}\right)$$

with constant maximum conductance ${\bar{g}}_{L}$ and Nernst potential $V_{L}$.

The model is thus described by a six-dimensional system representing the time-dependent membrane voltage V and the five time-dependent gating variables $m,n,n_{A},h_{A}$, and $h_{T}$.

### Model parameter values

Simulations of the model using parameter values listed in Molineux et al. (2005) and Anderson et al. (2010) produced cycles of APs in the (V,dV/dt)− plane that did not match the activities at either baseline (0 min) or the activities after the increase in excitability (data not shown). To capture the dynamics of the increase in excitability, we first fitted the expressions of $n_{A,\infty }$ and $h_{A,\infty }$ to the steady state activation/inactivation kinetic data of $I_{A}$ before and after the shift, then used the full model to manually fit its numerical simulations to baseline data before the increase in excitability. This was done in two sequential steps: (1) by first capturing all features of the AP cycles in the (V,dV/dt)− plane at baseline, followed by (2) matching the firing frequency obtained from the temporal profiles of membrane voltage. We will refer to this model associated with baseline data as the “baseline” model. To capture all features of the AP cycle after the increase in excitability (i.e., after 25 min), we identified the list of all parameters that need to be adjusted to produce these features in the (V,dV/dt)− plane and the higher firing frequency. Because our analysis revealed that the T-type Ca^2+^ current played a minor role in inducing the increase in excitability within the model, we left the kinetic parameters of activation/inactivation of $I_{T}$ listed in Molineux et al. (2005) and Anderson et al. (2010) unchanged for both before and after the increase in excitability. The reversal potentials of Na^+^, K^+^ and Ca^2+^ were also left unchanged. As for the remaining parameters, they were estimated based on fitting and parsimony. The resulting model that produced the increase in excitability will be referred to hereafter as “revised” model. The list of all parameter values for the baseline and revised models are provided in Table 1. Simulations were run in Mathematica 11.2 (Wolfram Mathematica, RRID:SCR_014448).

**Table 1.** Parameter values of ionic currents included in the baseline and revised models whenever two values are provided for a given parameter, the first corresponds to the baseline model (before the increase in excitability at baseline) while the second shown between parentheses corresponds to the revised model (after the increase in excitability at 25 min)

Parameter	Value/units	Parameter	Value/units	Parameter	Value/units	Parameter	Value/units
$C_{M}$	1.50148 μF/cm^2^	$V_{Ca}$	22 mV	w	46 mV	$v_{m_{T}}$	−50 mV
${\bar{g}}_{Na}$	3.4 μS/cm^2^	$V_{L}$	−38 mV	$V_{c}$	−74 mV	$s_{m_{T}}$	3 mV
${\bar{g}}_{K}$	9.0556 μS/cm^2^	$v_{m}$	−37 (−44) mV	$v_{n}$	−23 mV	$v_{h_{T}}$	−68 mV
${\bar{g}}_{A}$	15.0159 μS/cm^2^	$s_{M}$	3 mV	$s_{n}$	5 mV	$s_{h_{T}}$	3.75 mV
${\bar{g}}_{T}$	0.45045 μS/cm^2^	$v_{h}$	−40(−48.5) mV	$v_{n_{A}}$	−27(−41) mV	$\tau_{n_{A}}$	5 ms
${\bar{g}}_{L}$	0.07407 μS/cm^2^	$s_{h}$	4 mV	$s_{n_{A}}$	13.2 mV	$\tau_{h_{A}}$	10 ms
$V_{Na}$	55 mV	$y_{0}$	0.1 ms	$v_{h_{A}}$	−80(−96) mV	$\tau_{h_{T}}$	15 ms
$V_{K}$	−80 mV	A	322 ms mV	$s_{h_{A}}$	6.5(9.2) mV		

### Experimental design and statistical analysis

Paired *t* tests were used to compare electrophysiological data recorded at baseline and after 25 min (step-evoked AP frequency, AP threshold, AP cycle parameters, V_1/2_ values for $I_{Na}$). In the cases of $I_{A}$ and $I_{K}$ gating properties, a two-way mixed design ANOVA was used with Tukey’s *post hoc* test to compare patch configuration data (nucleated and whole-cell) between groups while comparing data over time (baseline and 25 min) within each group. For each experimental group, recordings from a minimum of five cells from a minimum of three animals were collected. Analysis was not blinded.

Voltage dependence of activation for all current types was analyzed by first calculating conductance (G) values from the peak currents elicited by each respective activation protocol using the formula:

$$G_{x}=\frac{I_{x}}{V_{m}-V_{rev_{x}}}$$

where $I_{x}$ is the peak current of current type x (i.e., $I_{A}$, $I_{K}$, $I_{Na}$) evoked at membrane potential $V_{m}$, and $V_{rev_{x}}$ is the reversal potential of current type x. Conductance-voltage relationships for each current type were then fit to a Boltzmann function using the formula:

$$G_{x}=\frac{G_{max}}{1+\text{exp}((V_{1/2}-V_{m})/k)}$$

where $G_{x}$ is the conductance at membrane potential $V_{m}$ for current type x, $G_{max}$ is the maximum conductance for current type x, $V_{1/2}$ is the membrane potential where $G_{x}$ is 50% of $G_{max}$, and k is the slope factor. Boltzmann fits were performed for each time point in each cell to calculate normalized conductance values. Summary conductance-voltage relationships for each current type were calculated by averaging the normalized conductance values across all cells in the dataset. V_1/2_ values reported in the Results section for each current type were calculated by fitting the normalized conductance averages.

Data are reported as mean ± SEM. Significance was denoted with *p < 0.05, **p < 0.01, and ***p < 0.001. All fitting was performed with Origin 7.0 (Microcal Origin, RRID:SCR_002815). Statistical analyses were performed using SPSS 17.0 (SPSS, RRID:SCR_002865).

## Results

### AP firing in cerebellar stellate and GC (granule cell), but not Purkinje cells, increases during whole-cell recording

**Figure 1.**
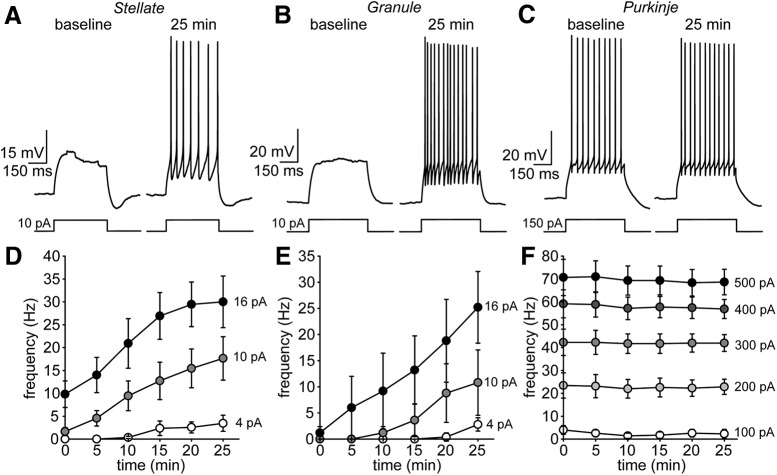
Stellate and GC (granule cell), but not Purkinje cells, exhibit excitability increases during whole-cell recording. ***A***, Example SC current-clamp recording (patch #150129p2) applying a 10-pA step protocol shortly following breakthrough and after 25 min. ***B***, ***C***, Same for GC (patch #150324p2) and Purkinje cell (patch #141010p5) examples using 10- and 150-pA current steps, respectively. ***D***, Summary SC AP frequency over 25-min recording for multiple step amplitudes (*n* = 11 cells). ***E***, ***F***, Same for granule (*n* = 6 cells) and Purkinje cells (*n* = 6 cells).

To examine time-dependent changes in membrane excitability, whole-cell patch-clamp recordings were obtained from three types of visually-identified cerebellar neurons, namely stellate, granule and Purkinje cells (Fig. 1*A–C*). After obtaining the whole-cell current-clamp configuration, negative current was injected into each neuron to maintain the membrane potential at –70 or –80 mV (see Materials and Methods). To assess membrane excitability following breakthrough, incremental depolarizing current steps were applied within the first minute (termed baseline), and then once every 5 min during a typical 25-min recording (Fig. 1*A–F*).

Under these conditions, both stellate and GCs fired many more APs at the 25-min time point (Fig. 1*D*,*E*). For example, in SCs, AP frequency evoked by a 16-pA current step at baseline was 9.8 ± 2.9 Hz versus 30.0 ± 5.6 Hz after 25 min (*t* = 4.73, *p* < 0.001; *n* = 11 cells; Fig. 1*A*,*D*). In contrast, firing rates in Purkinje cells remained stable throughout the recording (Fig. 1*C*,*F*) but required larger step depolarizations to elicit APs. For example, AP frequency evoked by a 400-pA step at baseline was 59.1 ± 6.1 Hz versus 56.9 ± 4.2 Hz after 25 min (*t* = –0.88, *p* = 0.41; *n* = 7 cells). The increase in AP frequency in SCs was not accompanied by a change in input resistance (1011 ± 148 MΩ at baseline vs 967 ± 137 MΩ at 25 min; *t* = –1.21, *p* = 0.26; *n* = 11 cells) or resting membrane potential (–50.6 ± 1.3 mV at baseline vs –52.2 ± 1.6 mV at 25 min; *t* = –1.33, *p* = 0.23; *n* = 7 cells) suggesting that the increase in membrane excitability was primarily due to changes in the activity of active membrane conductances (i.e., ion channels). In contrast, the increase in step-evoked AP frequency in GCs (16-pA step: 1.2 ± 1.2 Hz at baseline vs 25.2 ± 6.7 Hz at 25 min; *t* = 4.07, *p* = 0.015; *n* = 5 cells; Fig. 1*B*,*E*) was accompanied by a concurrent increase in membrane input resistance (44.5 ± 13.4% increase, *n* = 3 cells). Given this, we focused the rest of our study on SCs to pinpoint the molecular events that give rise to the increase in membrane excitability.

**Figure 2.**
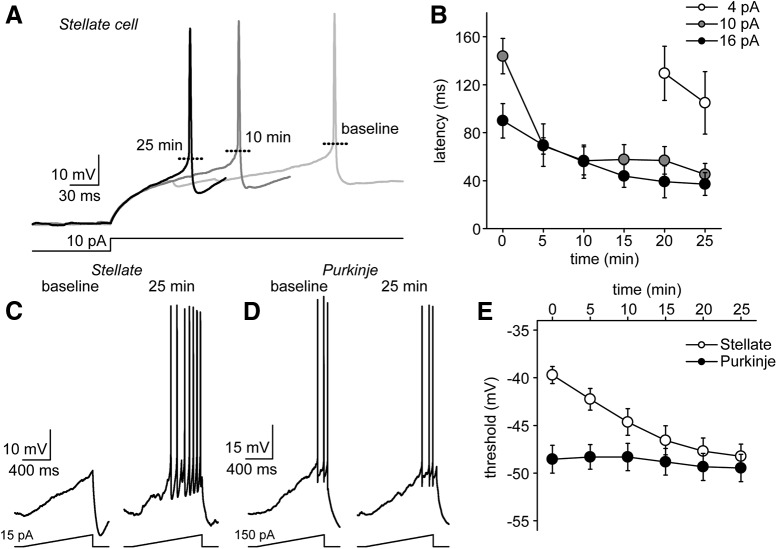
Excitability increase is underpinned by decrease in spike latency and hyperpolarization of AP threshold. ***A***, First APs fired by a SC in response to 10-pA current step at three different time points: 1, 10, and 25 min after breakthrough. The spike latency in response to the step decreases substantially over the course of the recording. ***B***, Summary plot of spike latencies for multiple step amplitudes over the course of a 25-min recording (*n* = 11 cells). ***C***, Example current-clamp responses evoked by ramp protocol at baseline (left) and after 25 min (right) in a SC (patch #150129p2). ***D***, Same for an example Purkinje cell (patch #141010p5). ***E***, Summary plot depicting change in AP threshold over time in stellate (*n* = 11 cells) and Purkinje cells (*n* = 6 cells).

A closer examination of SC recordings revealed a decrease in spike latency in response to current injection (Fig. 2*A*) at all current step amplitudes tested (Fig. 2*B*). For example, the latency to first spike in response to a 16-pA current step at baseline was 89.9 ± 14.4 versus 37.1 ± 9.5 ms at 25 min (*t* = –6.68, *p* < 0.001; *n* = 11 cells). To measure the shift precisely, we used a 1-s current ramp protocol to monitor the membrane potential at the initiation of the AP upstroke. To ensure that the ramp protocol had no direct effect on spike threshold, the protocol began with a 5-pA ramp that increased five times in increments of 5 pA. The threshold for each cell was then calculated by averaging the value obtained at each ramp amplitude. For comparison, we also repeated these experiments on both granule (not shown) and Purkinje cells (Fig. 2*D*,*E*). As anticipated, SCs exhibited a shift in AP threshold starting at –39.7 ± 0.9 mV following breakthrough and hyperpolarizing to –48.2 ± 1.3 mV at 25 min (*t* = –15.74, *p* < 0.001; *n* = 12 cells; Fig. 2*C*,*E*). GCs exhibited an even more substantial hyperpolarizing shift in AP threshold (–35.9 ± 0.6 mV at baseline vs –52.5 ± 2.1 mV at 25 min; *t* = –7.31, *p* < 0.001; *n* = 6 cells) whereas the threshold in Purkinje cells remained constant (–48.5 ± 1.5 mV at baseline vs –49.5 ± 1.4 mV at 25 min; *t* = –0.073, *p* = 0.51; *n* = 5 cells; Fig. 2*E*).

To ensure that the excitability increase in SCs was present in physiologically relevant conditions, the temperature of the aCSF perfusion was raised to near-physiologic level (33°C–34°C) and current-clamp experiments were repeated. SCs at elevated ambient temperatures still exhibited hyperpolarizing AP thresholds that closely resembled those at room temperature (–37.4 ± 1.7 mV at baseline vs –47.4 ± 1.9 mV at 20 min; *t* = 10.04, *p* < 0.001; *n* = 5 cells). Lastly, since others have noted that internal anions can affect intrinsic membrane properties of neurons during whole-cell recording (Kaczorowski et al., 2007), we repeated current-clamp experiments in SCs using a K-methanesulfonate-based intracellular solution in place of the initial K-gluconate version. Although slightly hyperpolarized at baseline compared to gluconate, the presence of methanesulfonate did not prevent the decrease of AP threshold over the duration of patch-clamp recording (–42.8 ± 1.1 mV at baseline vs –51.0 ± 1.8 mV at recording endpoint; *t* = 6.14, *p* < 0.001; *n* = 7 cells).

### Modified Hodgkin–Huxley model predicts a dominant role for voltage-gated Na^+^ channels

To better understand the change in excitability of SCs, we used a modified Hodgkin–Huxley model to examine the potential impact of ion channels that are likely to be involved. The model was based on a previous study that included a voltage-gated Na^+^ current ($I_{Na}$), a delayed rectifier K^+^ current ($I_{K}$), an A-type K^+^ current ($I_{A}$), a T-type Ca^2+^ current ($I_{T}$) and a leak current ($I_{L}$; Molineux et al., 2005). The A-type K^+^ and T-type Ca^2+^ currents were specifically included in the model as they have been shown to play key roles in determining first spike latencies (Molineux et al., 2005; Anderson et al., 2010, 2013). The model parameters were then adjusted to match observations of spontaneous AP firing at the beginning of SC recordings (see Materials and Methods).

**Figure 3.**
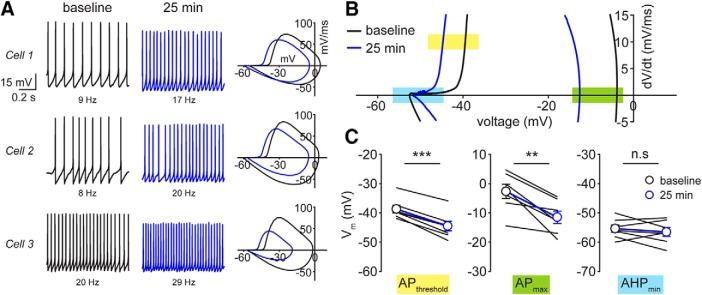
Defining the hallmark membrane features of SC excitability increase. ***A***, left panels, Spontaneous AP firing traces from three example SCs at baseline (black) and after 25 min (blue; patch #150129p2, #181111p6, #181109p3). Right panels, AP cycles for each cell calculated from voltage-time data at baseline (black) and 25 min (blue). ***B***, AP cycle plot of an example SC with *y*-axis expanded. Colored rectangles designate quantities of interest (yellow, AP threshold; green, AP maximum; light blue, AHP minimum). ***C***, Summary comparison of these three features measured from individual AP cycles (*n* = 7 cells). ^***^ denotes *p* < 0.001, ^**^ denotes *p* < 0.01, n.s denotes *p* > 0.05.

**Figure 4.**
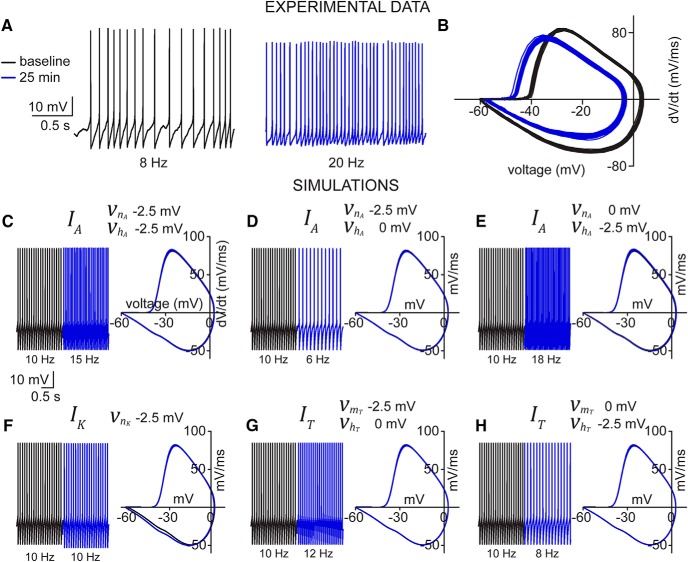
Simulating $I_{A}$, $I_{K}$, and $I_{T}$ shifts predicts little contribution to excitability increase. ***A***, Example SC current-clamp recording depicting spontaneous AP firing at baseline (black) and after 25 min (blue; patch #181111p5). ***B***, AP cycle calculated from current-clamp data from ***A*** at baseline (black) and 25 min (blue). ***C–E***, Simulated spontaneous firing and AP cycle from baseline model (black) and after making symmetric and asymmetric shifts in $v_{n_{A}}$ and $v_{h_{A}}$(blue). ***F***, Same for $I_{K}$ after shifting $v_{n_{K}}$(blue). ***G***, ***H***, Same for $I_{T}$ after shifting either $v_{m_{T}}$ or $v_{h_{T}}$.

To provide an accurate experimental baseline to use as a template for modeling spontaneous AP firing, gap-free recordings from multiple SCs were made in the absence of injected current (Figs. 3, 4*A*). In line with the previous current step and ramp experiments, spontaneous AP firing in all SCs increased significantly over 25 min (12.5 ± 3.2 Hz at baseline vs 17.9 ± Hz at 25 min; *t* = 2.60, *p* = 0.041; *n* = 7 cells; Figs. 3*A*, 4*A*). The AP cycle, which was obtained from these data by taking the derivative of the measured voltage plotted against membrane potential (Figs. 3*A*, 4*B*), revealed two defining characteristics of time-dependent changes in AP shape: (1) a negative shift in AP upstroke, indicating the hyperpolarization of spike threshold (defined as dV/dt = 10 mV/ms; Fig. 3*B*, yellow box); (2) a reduction in the peak of the AP cycle where it intersects with the voltage-axis (Fig. 3*B*, green box). These measurements were compared over multiple SCs, demonstrating significant differences in AP threshold (–38.7 ± 1.3 mV at baseline vs –44.5 ± 1.7 at 25 min; *t* = –11.25, *p* < 0.001; *n* = 7 cells; Fig. 3*C*) as well as AP maximum (–2.7 ± 2.4 mV at baseline vs –11.5 ± 2.1 mV at 25 min; *t* = –4.41, *p* = 0.0045; *n* = 7 cells; Fig. 3*C*). The change in after-hyperpolarization (AHP) minimum (Fig. 3*B*, light blue box) was variable across cells (three examples in Fig. 3*A*), but this was not significant (–55.4 ± 1.3 mV at baseline vs –56.5 ± 1.5 mV at 25 min; *t* = –0.88, *p* = 0.41; *n* = 7 cells; Fig. 3*C*). Together, these findings identify three cardinal features that are observed during whole-cell recording from SCs: (1) an increase in spontaneous AP frequency, (2) a hyperpolarizing shift of AP threshold, and (3) a hyperpolarizing shift in the AP maximum.

To examine the potential impact of each ionic current, we systematically modified their activation and inactivation kinetic parameters in the baseline model and compared the resulting AP cycle with experimental data. For example, a symmetrical negative shift of both activation ($n_{A,\infty }$) and inactivation ($h_{A,\infty }$) functions of $I_{A}$, obtained by decreasing both $v_{n_{A}}$ and $v_{h_{A}}$ by –2.5 mV, yielded a ∼50% increase in AP firing frequency but no change in the shape of the AP cycle (Fig. 4*C*). Likewise, shifts in either activation (Fig. 4*D*) or inactivation (Fig. 4*E*) functions of $I_{A}$ separately showed substantial shifts in firing frequency without altering the AP cycle shape or threshold. A negative shift in the activation function ($n_{\infty }$) of $I_{K}$, obtained by decreasing $v_{n}$ by –2.5 mV, resulted in no change in AP firing frequency but produced a leftward extension of the AHP in the AP cycle (Fig. 4*F*). Shifting either the activation function $m_{T,\infty }$(Fig. 4*G*) or inactivation function $h_{T,\infty }$(Fig. 4*H*) of $I_{T}$, obtained by decreasing each $v_{m_{T}}$ and $v_{h_{T}}$ separately by –2.5 mV, had little effect on AP firing frequency (where only an ∼20% increase in the former and a ∼20% decrease in the latter were observed) and no effect on the AP cycle. Given this, we concluded that T-type Ca^2+^ channels are unlikely to contribute to the temporal increase in excitability of SCs. Although the A-type K^+^ channel may contribute to the increase in AP firing rates, other ion channels must be responsible for the hyperpolarizing shift in AP threshold.

**Figure 5.**
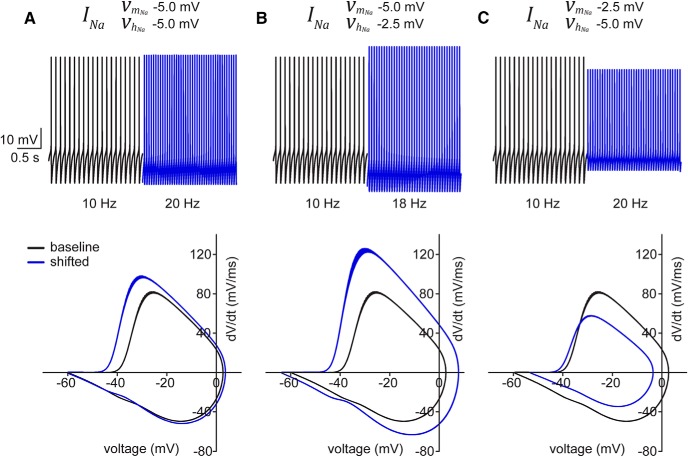
Simulated $I_{\mathrm{Na}}$ shifts suggest primary role for sodium channel in excitability increase. Simulated spontaneous firing (upper panels) and AP cycles (lower panels) from baseline model (black) and after either symmetric (***A***) or asymmetric (***B***, ***C***) shifts in $v_{m_{Na}}$ and $v_{h_{Na}}$.

Modification of the model parameters defining $I_{Na}$ had a substantial impact on AP firing and the AP cycle profile (Fig. 5). Identical negative shifts in both activation and inactivation parameters resulted in a concomitant shift in the AP cycle upstroke as well as an increase in AP firing frequency, although a reduction in the AP maximum was absent (Fig. 5*A*). Likewise, a differential left-shift in favor of the activation function (given by –5 mV for activation and –2.5 mV for inactivation) produced a ∼80% increase in AP firing frequency, but was unable to generate a reduction in AP maximum (Fig. 5*B*). In contrast, a differential left-shift in favor of inactivation resulted in a ∼100% increase in AP firing as well as reproducing the profile of the AP cycle similar to that observed experimentally (Fig. 5*C*). Taken together, the model suggests that hyperpolarizing shifts in the gating properties of $I_{Na}$ alone can account for the increase in firing rate as well as the characteristic AP cycle changes exhibited by SCs during patch-clamp recording.

### SC excitability is accompanied by gating shifts in $I_{A}$ but not $I_{K}$

**Figure 6.**
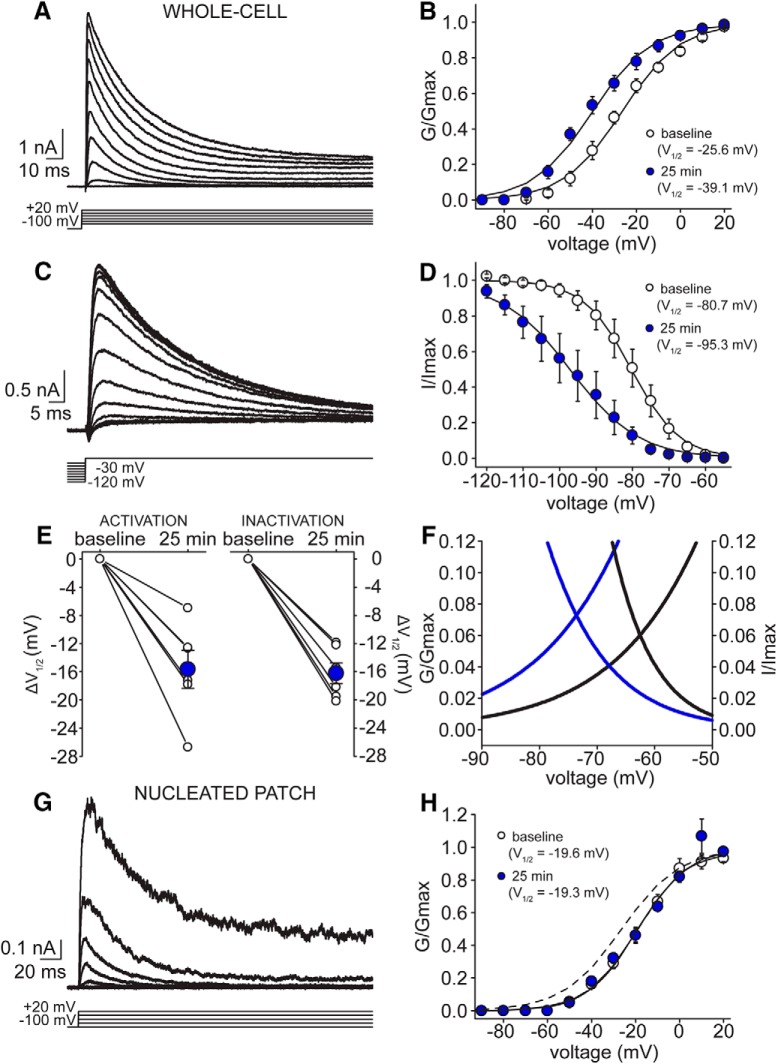
$I_{A}$ exhibits shifts in both activation and inactivation during 25-min recording. ***A***, Example voltage-clamp traces of $I_{A}$ currents at baseline during activation protocol (patch #171024p3). ***B***, Summary plot of voltage dependence of activation of $I_{A}$ at baseline (white circles) and after 25 min of recording (blue circles; *n* = 7 cells). ***C***, Example voltage-clamp traces of $I_{A}$ currents evoked during inactivation protocol (patch #171127p3). ***D***, Summary plot of voltage dependence of inactivation of $I_{A}$ at baseline (white circles) and after 25 min of recording (blue circles; *n* = 7 cells, same as in ***B***). ***E***, Normalized V_1/2_ activation (left) and inactivation (right) compared to delta shift after 25-min recording for each cell (white circles), along with summary mean delta for each measure (blue circles). ***F***, Zoom-in of Boltzmann fits for both voltage dependence of activation and inactivation at baseline (black lines) and at 25 min (blue lines) from ***B***, ***D***, respectively, depicting symmetrical translocation of $I_{A}$ window current. ***G***, Example voltage-clamp traces of $I_{A}$ currents during activation protocol observed in a SC nucleated patch (patch #180510p3). ***F***, Summary plot of voltage dependence of activation of $I_{A}$ at baseline (white circles) and 25 min (blue circles; *n* = 6 patches). Dashed line depicts baseline activation curve measured in whole-cell configuration from ***B***.

**Figure 7.**
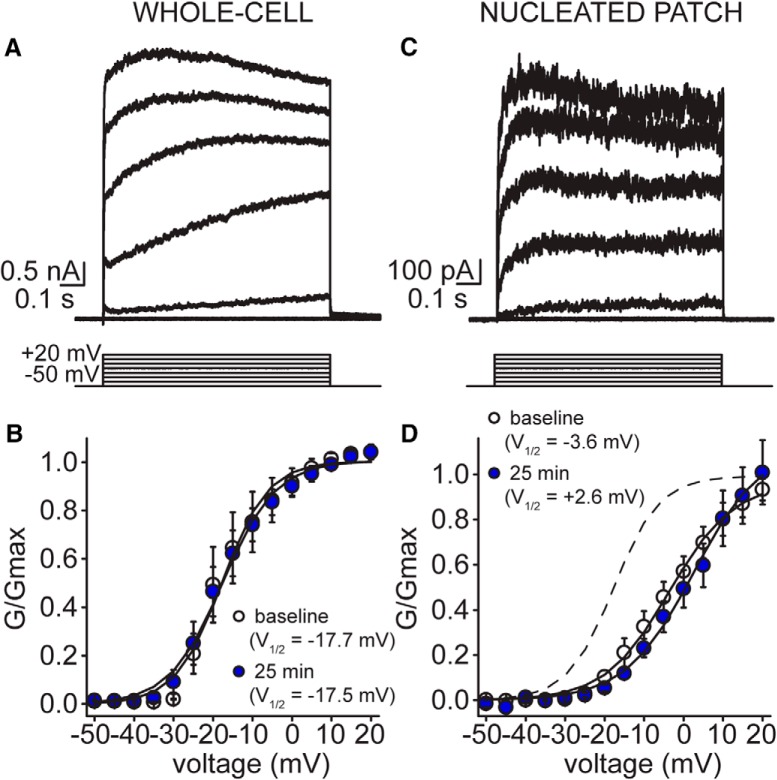
$I_{K}$ voltage dependence of activation remains stable over patch-clamp recording in both whole-cell and nucleated patch configurations. ***A***, Example whole-cell voltage-clamp traces of delayed rectifier K^+^ current at baseline during activation protocol evoked from –50-mV holding potential (5-mV increments, up to +20 mV; patch #180521p2). ***B***, Summary plot of voltage dependence of activation of delayed rectifier K^+^ current at baseline (white circles) and after 25 min of recording (blue circles; *n* = 8 cells). ***C***, Example voltage-clamp traces after excising a nucleated patch of delayed rectifier K^+^ current at baseline during activation protocol evoked from –50-mV holding potential (5-mV increments, up to +20 mV; patch #180510p3). ***D***, Summary plot of voltage dependence of activation of delayed rectifier K^+^ current at baseline (white circles) and after 25 min of recording (blue circles; *n* = 6 cells). Dashed line depicts baseline activation curve measured in whole-cell configuration from ***B***.

To directly test the prediction of the model, we used customized activation and inactivation voltage-clamp protocols to isolate two subtypes of K^+^ current, $I_{A}$ and $I_{K}$ (Figs. 6, 7; see Materials and Methods). Since it can be problematic to accurately measure voltage-gated ion channel activity in highly ramified structures such as neurons, we repeated experiments in nucleated patches where a more faithful voltage-clamp control can be achieved. In whole-cell recordings, we observed a significant time-dependent shift in the voltage dependence of both activation and inactivation of $I_{A}$ (Fig. 6*B*,*D*). From Boltzmann fits of conductance-voltage relationships, V_1/2_ activation of $I_{A}$ shifted from –25.8 ± 2.4 mV at baseline to –39.1 ± 2.6 mV after 25 min (*F* = 7.07, *p* < 0.001, Tukey’s *post hoc* test; *n* = 7 cells), while V_1/2_ inactivation changed from –80.7 ± 2.8 mV at baseline to –95.3 ± 4.6 mV at 25 min (*t* = –11.91, *p* < 0.001; *n* = 7 cells). Plotting the normalized changes in V_1/2_ measurements between baseline and after 25 min for individual cells yielded a comparable result (ΔV_1/2_ activation: –15.7 ± 2.7 mV; ΔV_1/2_ inactivation: –16.2 ± 1.5 mV; Fig. 6*E*). Since the changes in V_1/2_ activation and V_1/2_ inactivation are very similar, this produced a hyperpolarizing shift in the window current of about –11 mV with the midpoint moving from –62.5 to –73.5 mV (Fig. 6*F*). Interestingly, the shift in the voltage dependence of activation of $I_{A}$ was completely lost in nucleated patches suggesting that the time-dependent change in the gating properties of $I_{A}$ is probably mediated by a cytoplasmic signaling pathway, such as phosphorylation (Fig. 6*G*,*H*). The V_1/2_ activation was –19.6 mV at baseline and –19.3 mV after 25 min of recording (*F* = 0.07, *p* = 0.96, Tukey’s *post hoc* test; *n* = 6 patches), which was slightly more depolarized than measurements in whole-cell recordings (*F* = 3.31, *p* = 0.038, Tukey’s *post hoc* test; Fig. *6H*), possibly reflecting the improvement in voltage-clamp control.

In contrast to $I_{A}$, there was no significant time-dependent change in the voltage dependence of $I_{K}$ activation in whole-cell recording, nor in nucleated patches (*F* = 3.88, *p* = 0.081, two-way mixed design ANOVA; Fig. 7*A–D*). In whole-cell configuration, V_1/2_ activation at baseline was –17.7 mV compared to –17.5 mV at 25 min (*n* = 8 cells), while the voltage dependence of activation in excised patches (*n* = 6) was –3.6 mV at baseline compared to +2.6 mV at 25 min. There was, however, a significant difference in V_1/2_ values between measurements made in whole-cell and nucleated patches (*F* = 23.42, *p* < 0.001, two-way mixed design ANOVA), suggesting that the recording configuration (whole-cell vs patch) affects $I_{K}$ gating.

### $I_{Na}$ exhibits hyperpolarizing shifts in both activation and inactivation

**Figure 8.**
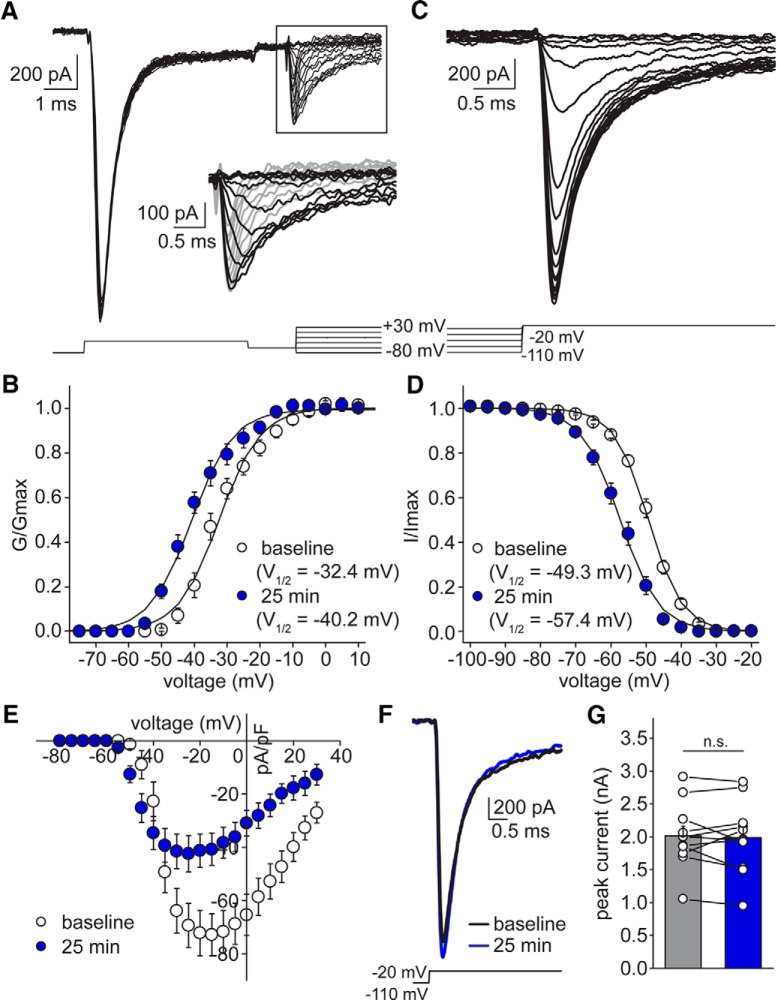
Na^+^ current SCs exhibits shift in both activation and inactivation properties, but not current density. ***A***, Example voltage-clamp traces of Na^+^ currents evoked with a prepulse protocol (patch #170222p4). Inset, Zoom-in of portion of traces depicting strong voltage-clamp of Na^+^ currents evoked after prepulse. ***B***, Summary plot of voltage dependence of activation of Na^+^ current at baseline (white circles) and at 25 min (blue circles; *n* = 11 cells). ***C***, Example traces from a Na^+^ inactivation protocol (patch #170222p4). ***D***, Summary plot of voltage dependence of inactivation at baseline (white circles) and at 25 min (blue circles; *n* = 11 cells). ***E***, Summary current density-voltage plot (data used to construct conductance-voltage plots in ***B***, ***D***, of sodium responses at baseline (white circles) and at 25 min (blue circles). ***F***, Example peak sodium current traces evoked by a –110 to –20 mV probe at baseline (black) and at 25 min (blue; patch #171108p3). ***G***, Summary graph of peak sodium currents evoked from a holding potential of –110 mV at baseline (gray) and at 25 min (blue). n.s. denotes *p* > 0.05.

Because of space clamp issues, it is not possible to record the fast gating of $I_{Na}$ in SCs with conventional voltage-clamp protocols. The main problem is the inability to accurately record Na^+^ channel activity in distant processes such as axons. To circumvent this, we adapted a protocol using a depolarizing prepulse step to inactivate Na^+^ channels distant from the recording electrode which was followed shortly afterward with a second test step to record Na^+^ channels close to the cell soma (Milescu et al., 2010). Using this approach, we reliably resolved well-clamped, albeit smaller, $I_{Na}$ responses in SCs to profile their activation and inactivation properties (Fig. 8*A–D*). Performing this protocol over a 25 min period revealed that SC Na^+^ channels undergo a hyperpolarizing shift of –7.8 mV in their activation (–32.4 ± 1.5 mV at baseline vs –40.2 ± 1.6 mV at 25 min; *t* = –9.10, *p* < 0.001; *n* = 11 cells; Fig. 8*A*,*B*) and a similar hyperpolarizing shift in steady-state inactivation (–49.3 ± 0.7 mV at baseline to –57.4 ± 1.0 mV at 25 min; *t* = –8.95, *p* < 0.001; *n* = 11 cells; Fig. 8*C*,*D*). This finding is in agreement with the hyperpolarizing shift in Na^+^ channel gating predicted by the modified Hodgkin–Huxley model applied to stellate AP firing (Fig. 5).

Interestingly, we also observed a reduction in peak $I_{Na}$ current density that developed over the duration of the experiment (–75.5 ± 8.4 pA/pF at baseline vs –45.1 ± 6.7 pA/pF at 25 min; *t* = 6.64, *p* < 0.001; *n* = 11 cells; Fig. 8*E*). To test whether this was due to a reduction in $I_{Na}$ current density or a consequence of the hyperpolarizing shift in inactivation, we compared peak currents from the first step of the inactivation protocol (–110- to –20-mV probe; Fig. 8*F*). At this voltage, all SC Na^+^ channels are available for activation both at baseline and after a 25-min recording. According to this measure, there is no difference between Na^+^ current density before and after 25 min and this reduction reflects the shift in steady-state inactivation alone (peak current: –2012.3 ± 149.1 pA at baseline vs –1984.5 ± 162.8 pA at 25 min; *t* = 0.43, *p* = 0.68; *n* = 11 cells; Fig. 8*F*,*G*).

### Pharmacological block of K^+^, Ca^2+^, or HCN channels has no effect on AP threshold

**Figure 9.**
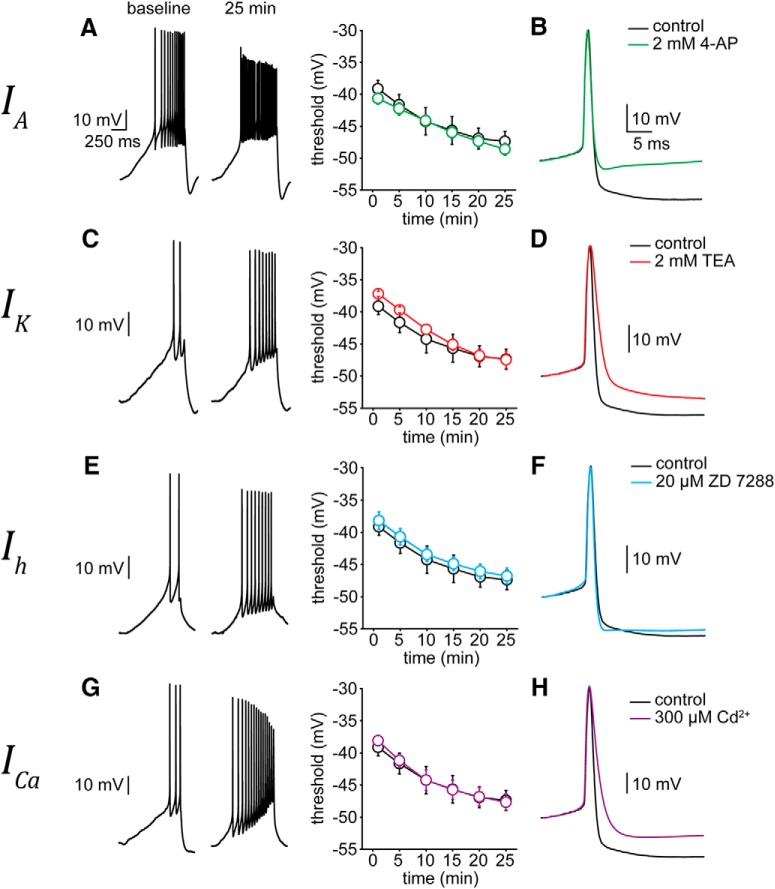
AP threshold hyperpolarization persists with pharmacological blockade of $I_{K}$, $I_{A}$, $I_{\mathrm{Ca}}$ and $I_{h}$. ***A***, left panel, Example traces recorded at baseline and after 25 min of APs evoked by a current ramp protocol (25 pA over 1 s) in a SC. Right panel, Summary AP threshold data for control conditions (black open circles; *n* = 6 cells) and in the presence of 2 mM TEA in the aCSF (red open circles; *n* = 5 cells). ***B***, Superimposed first APs evoked by ramp protocol in control (black) and external TEA (red) conditions. TEA scaled to control (patch #181008p8). ***C***, ***D***, Same as ***A***, ***B***, but in the presence of 2 mM 4-AP (green lines; *n* = 5 cells; patch #181015p7). ***E***, ***F***, Same as ***A***, ***B***, but in the presence of 20 µM ZD 7288 (blue lines; *n* = 6 cells; patch #181018p2). ***G***, ***H***, Same as in ***A***, ***B***, but in the presence of 200–300 µM CdCl_2_ (purple lines; *n* = 5 cells; patch #181024p4).

To test the hypothesis that the shift in Na^+^ channel gating is primarily responsible for the increase in SC excitability, we examined whether pharmacological block of $I_{A}$ with 4-AP attenuates the hyperpolarizing shift in AP threshold. As expected, block of $I_{A}$ with bath application of 2 mM 4-AP resulted in a more depolarized inter-spike membrane potential at baseline compared to control cells (Fig. 9*B*). 4-AP, however, did not attenuate the shift in AP threshold (Fig. 9*A*) which hyperpolarized from –40.7 ± 0.9 mV at baseline to –48.6 ± 0.9 mV after 25 min (*t* = –9.37, *p* < 0.001; *n* = 5 cells). Interestingly, 2 mM 4-AP also attenuated the AHP of the AP (Fig. 9*B*) which may reflect pharmacological block of delayed rectifier K^+^ channels. In keeping with this, block of $I_{K}$ with 2 mM external tetraethylammonium (TEA) had the expected effect on AP shape at baseline but did not attenuate the shift in AP threshold (Fig. 9*C*,*D*). AP threshold observed in 2 mM TEA hyperpolarized from –37.2 ± 0.4 mV at baseline to –47.5 ± 1.4 mV after 25 min of recording (*t* = –8.99, *p* < 0.001; *n* = 5 cells; Fig. 9*C*,*D*). Since hyperpolarization-activated cyclic nucleotide-gated (HCN) channels (which mediate the mixed cation current $I_{h}$) and voltage-gated Ca^2+^ channels (VGCCs) have also been shown to affect excitability of molecular layer interneurons (MLIs; Saitow and Konishi, 2000; Anderson et al., 2010, 2013), we tested the effect of the selective $I_{h}$ blocker, ZD 7288 (20 µM) and nonselective VGCC blocker CdCl_2_ (200 – 300 µM) on AP threshold (Fig. 9*E–H*). In each case, the hyperpolarizing shift in AP threshold was unaffected by either ZD 7288 or CdCl_2_. The AP threshold with 20 µM ZD 7288 was –38.2 ± 1.3 mV at baseline and hyperpolarized to –46.8 ± 1.3 mV at 25 min (*t* = –10.13, *p* < 0.001; *n* = 6 cells). Likewise, the threshold shifted from –38.1 ± 0.8 mV at baseline with CdCl_2_ to –47.7 ± 1.3 mV after 25 min of recording (*t* = –7.69, *p* = 0.0015; *n* = 5 cells). Taken together, these data demonstrate that $I_{A}$_,_ $I_{K}$, $I_{h}$ and VGCCs do not contribute to the hyperpolarization of AP threshold of SCs, consistent with our hypothesis that the primary mechanism for the shift is mediated by Na^+^ channels.

### Revised Hodgkin–Huxley model recapitulates the increase in SC excitability

**Figure 10.**
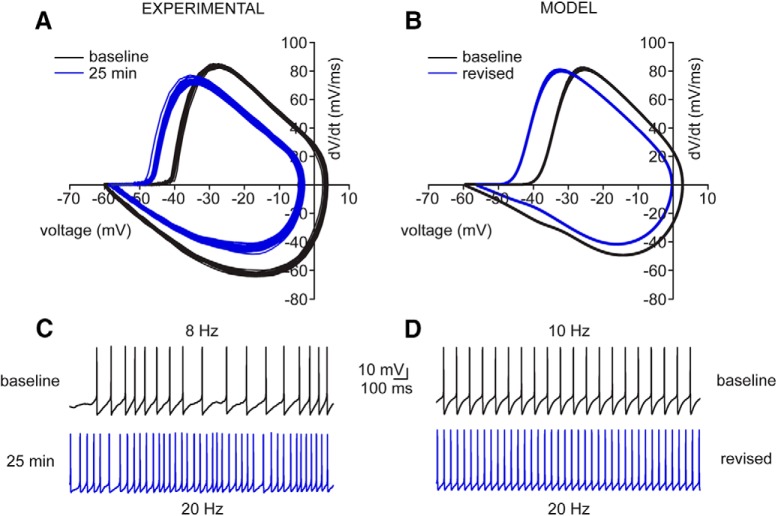
Voltage-clamp-informed model recapitulates the dynamics of current-clamp experimental data. ***A***, AP cycle calculated from experimental SC current-clamp recording at baseline (black) and 25 min (blue). ***C***, Same current-clamp recording data as above, presented as voltage versus time at baseline (black) and 25 min (blue). ***B***, Simulated AP cycle of baseline (black) and revised (blue) models after modifying all gating parameters based on values measured experimentally in voltage-clamp in SCs. ***D***, Simulated firing properties of baseline (black) and revised (blue) models.

Based on the voltage-clamp data, we revised the Hodgkin–Huxley model to match the observed changes in $I_{Na}$ and $I_{A}$ (see Materials and Methods). To do this, we targeted only their activation/inactivation properties without altering their conductances. Specifically, $v_{m_{Na}}$ was shifted by –7 mV and $v_{h_{Na}}$ by –8.5 mV, while $v_{n_{A}}$ was shifted by –14 mV and $v_{h_{A}}$ by –16 mV (Table 1), which reproduced well the observed increase in excitability of SCs (Fig. 10*A–D*). With respect to the changes in the AP cycle described earlier (Fig. 3), the revised model captures all the cardinal features seen in the experimental recordings (Fig. 10*A*,*B*). Specifically, the AP threshold of the revised model hyperpolarized by –5.8 mV from –38.7 to –44.5 mV, which agrees well with the –6.0-mV hyperpolarization observed experimentally. The simulated AP maximum hyperpolarized by –8.4 mV from 3.9 to –4.5 mV, which is comparable to the hyperpolarization of –8.8 mV measured experimentally. Finally, the AHP minimum observed in the revised model depolarized by +1.8 mV from –60.1 to –58.3 mV, which is within the margin of error of the experimental results (Fig. 3*C*). The fact that these specific adjustments are able to closely capture the dynamics observed experimentally further supports the dominance of $I_{Na}$ in driving the increase in SC excitability, with an additional role for $I_{A}$ in further modulating AP firing.

## Discussion

The present study advances our understanding of the neurophysiology of cerebellar SCs in two important ways. First, we identify a predominant role of voltage-gated Na^+^ channels in upregulating the excitability of cerebellar SCs following membrane patch breakthrough. Under these conditions, the threshold for AP initiation is shifted to more hyperpolarized membrane potentials due to changes in the activation and inactivation properties of Na^+^ channels. Since similar shifts in gating behavior are observed in other central neurons by physiologic stimuli, such as NMDA receptor activation (Xu et al., 2005), it is possible that Na^+^ channels are targeted in a comparable manner in SCs to upregulate AP firing. Second, we report hyperpolarizing shifts in both the activation and inactivation properties of A-type K^+^ channels that contribute to the time-dependent increase in firing rates observed in SCs but do not participate in the hyperpolarizing shift in AP threshold. Previous work has established that depolarizing shifts in A-type K^+^ channel inactivation are triggered by elevations in cytosolic Ca^2+^ due to T-type Ca^2+^ channel activity in SCs (Molineux et al., 2005; Anderson et al., 2010, 2013). Given this distinction with the present study, it is likely that different signaling events are at play to regulate the gating behavior of A-type K^+^ channels in cerebellar SCs.

### Increased membrane excitability following whole-cell recording is not unique to SCs

SCs are not the only neuronal cell type that undergo functional changes after the establishment of the whole-cell patch-clamp configuration. In fact, early patch clamp studies of voltage-gated ion channels noted time-dependent changes in both activation and inactivation properties including changes to voltage-gated Na^+^ channels (Fenwick et al., 1982; Fernandez et al., 1984; Vandenberg and Horn, 1984; Marty and Neher, 1985). The mechanism(s) underlying these changes has remained largely unexplored and thus ignored - as has been the case for cerebellar SCs. In the cerebellum, the increased firing rate we have observed in GCs (compare Fig. 1) may be attributed to the cell’s buffering capacity for cytosolic Ca^2+^ which is further compromised when GCs lack the calcium binding protein, calretinin (Gall et al., 2003). Similarly, Alcami and colleagues also linked a rise in intracellular Ca^2+^ to an increase in firing rates of SCs (Alcami et al., 2012) suggesting that there may be overlapping signaling pathways at play in both cell types (see below). A distinction with SCs, however, is that we have observed an increase in the input resistance of GCs following patch breakthrough. Whether the change in membrane leak is due to the activity of other ion channels, such as TASK-1 (Millar et al., 2000) or HCN channels (Zúñiga et al., 2016), in GCs remains to be studied. Why the excitability of SCs and GCs increases after patch breakthrough whereas Purkinje cells remain stable is not clear. It is tempting to speculate that the smaller soma of SCs makes their subcellular Ca^2+^ stores more susceptible to perturbation during whole-cell recording compared to Purkinje cells. It is also possible that SCs are mechanosensitive which is in keeping with the observation that SC excitability increases during cell-attached recordings, particularly in recordings with a tight seal (Alcami et al., 2012). As discussed below, the events that are initiated by membrane patch breakthrough suggest that voltage-gated Na^+^ channels may be impacted by several endogenous signaling pathways in SCs.

### Expression and regulation of Na^+^ channels in cerebellar SCs

Cerebellar MLIs are thought to express several Na^+^ channel pore-forming subunits that include Nav1.1, Nav1.2, and Nav1.6 (Schaller and Caldwell, 2003); however, more recent data suggest that the Nav1.2 protein is expressed only on presynaptic terminals of GCs (Jarnot and Corbett, 2006; Martínez-Hernández et al., 2013). This latter finding agrees with our experiments using the Nav1.2-selective inhibitor, Phrixotoxin-III, which has negligible pharmacological effect on SC excitability (Alexander and Bowie, unpublished observation). Based on antibody staining, both Nav1.1 and Nav1.6 subunits are expressed in MLIs (Kalume et al., 2007); however, their subcellular distribution is distinct (Lorincz and Nusser, 2008). The Nav1.6 subunit is almost exclusively expressed at the axon initial segment (AIS), a region of the axon close to the cell body where APs are initiated. In contrast, Nav1.1 is expressed throughout the axon, although it is also found in the proximal AIS (Lorincz and Nusser, 2008). Given this arrangement, both Nav1.1 and Nav1.6 subunits may contribute to the Na^+^ channel currents we have recorded from SCs. Whether these subunits are also expressed in the dendrites has not be formally investigated, although SC dendrites are apparently devoid of Na^+^ channels (Myoga et al., 2009). It is curious that the expression pattern of the delayed rectifier K^+^ channel subunits, Kv1.1 and Kv1.2, overlap with Nav1.6 subunits, but yet, are not subject to modulation following patch breakthrough (compare Fig. 9). This observation suggests that the signaling pathway(s) that promotes gating shifts in Na^+^ channels is tightly compartmentalized from other ion channels, such as Kv1.1 and Kv1.2, even within the AIS.

Gating shifts in Na^+^ channel activation and inactivation, as observed in the present study (compare Fig. 8), may, in principle, be mediated by a number of mechanisms that include direct binding by cytosolic Ca^2+^, binding of the Ca^2+^-calmodulin complex and/or changes in the phosphorylation state of the Na^+^ channel (Cantrell and Catterall, 2001; Van Petegem et al., 2012). Regulation of recombinant Nav1.6 channels has primarily focused on the effect of channel phosphorylation where protein kinase A (PKA) stimulation (Chen et al., 2008) and p38 mitogen-activated protein kinase (Wittmack et al., 2005) both cause a reduction in peak Na^+^ channel current. In the latter case, the reduction in current density did not affect channel gating (Wittmack et al., 2005), unlike the present study (compare Fig. 8) but was due to the internalization of Nav1.6 channels (Gasser et al., 2010). In contrast, inhibition of glycogen-synthase kinase 3β (GSK3β) causes a decrease in recombinant Nav1.6 responses (Scala et al., 2018) suggesting that phosphorylation sustains channel activity in HEK 293 cells. Likewise, in medium spiny neurons of the nucleus accumbens, regulation of GSK3β activity impacts firing rates in a Nav1.6-dependent manner (Scala et al., 2018) suggesting that phosphorylation and dephosphorylation events may be critical in fine-tuning neuronal output. Much less is known about the regulation of Nav1.1 channels, although, earlier recombinant studies and more recent proteomic work has identified putative phosphorylation sites which often overlap with sites identified for Nav1.2 channels (Smith and Goldin, 1998; Berendt et al., 2010). Interestingly, forskolin activation of PKA causes a hyperpolarizing shift in both the activation and inactivation curves for recombinant Nav1.1 (Liu and Zheng, 2013) as observed in the present study on cerebellar SCs. An identical gating shift is observed in hippocampal CA1 pyramidal cells, probably mediated by Nav1.2 channels, that is triggered by NMDA receptor activation and the activity of CaM kinase II (Xu et al., 2005). Given the similarity in how Nav1.1 and Nav1.2 channels are regulated by phosphorylation, it would be interesting to test if a NMDA receptor-mediated signaling pathway could induce a similar shift in the gating behavior of Na^+^ channels in cerebellar SCs. Likewise, experiments using dynamic clamp to test the effect of a Nav channel gating model on SC excitability would be interesting to test in future studies.

### Multiple families of voltage-gated ion channels control SC excitability

Although SCs express a variety of voltage-gated ion channels, somatodendritic A-type K^+^ channels are an important regulator of their firing rates (Molineux et al., 2005; Anderson et al., 2010, 2013). Specifically, AP firing rates are regulated by Kv4.2/4.3 subunits whose basal activity is fine-tuned by elevations in cytosolic Ca^2+^ mediated by the Ca^2+^ channel subunits, Cav3.2 and Cav3.3 (Anderson et al., 2010). Both ion channel families form a signaling complex through the modulatory protein, KChIP3, which contains an E-F hand and thus is able to act as a Ca^2+^ sensor that couples the activity of Ca^2+^ channels to the regulation of K^+^ channels (Pongs and Schwarz, 2010). Elevations in intracellular Ca^2+^ selectively promote depolarizing shifts in steady-state inactivation of A-type K^+^ channels and, in doing so, attenuate membrane depolarization and AP firing (Anderson et al., 2010, 2013). In contrast, our data establish that A-type K^+^ channels contribute to an increase in AP firing of SCs via a hyperpolarizing shift in channel activation and inactivation (compare Fig. 6). Although the formation of the whole-cell recording is likely to elevate cytosolic Ca^2+^ in SCs, as discussed above, the shifts A-type K^+^ channel gating observed in this study are probably not reliant on KChIP3 modulation. Finally, although other voltage-gated ion channels may be involved in controlling SC excitability, pharmacological block of many of these channels (Fig. 9) reveals that they do not impact AP threshold. Consequently, our work identifies a predominant role of voltage-gated Na^+^ channels in upregulating the firing rates of cerebellar SCs.

## Synthesis

Reviewing Editor: Zoltan Nusser, Institute of Experimental Medicine Decisions are customarily a result of the Reviewing Editor and the peer reviewers coming together and discussing their recommendations until a consensus is reached. When revisions are invited, a fact-based synthesis statement explaining their decision and outlining what is needed to prepare a revision will be listed below. The following reviewer(s) agreed to reveal their identity: Bruce Bean. Note: If this manuscript was transferred from JNeurosci and a decision was made to accept the manuscript without peer review, a brief statement to this effect will instead be what is listed below.

In the present manuscript Alexander and colleagues investigate the mechanisms involved in the time-dependent alteration in cerebellar stellate cell excitability induced by the whole-cell configuration. The authors observe that the increase in cell excitability results from a hyperpolarizing shift in the gating behaviour of voltage-gated Na+ channels leading to a lower membrane potential at which APs are initiated. The authors indicate that whole cell recordings induce also a hyperpolarizing shift in the gating behaviour of A-type K+ channels which contributes to increased firing rates. Hodgkin-Huxley modelling nicely supports authors conclusions.

The work is well performed and data of high quality. However, even if the authors performed an important set of experiments to carefully identify the origin of the time-dependent increase in SC excitability while in whole-cell mode, no clear solution is presented to minimize such an increase. I don't expect the authors to find the solution for this modification in cell excitability but testing one or two possibilities, some of them even raised by the authors in the discussion, could be extremely useful for potential readers of the present work.

Major points:

1- The authors mention the washout of the endogenous calcium buffer calretinin in GC, but the same is true for Parvalbumin (which endogenous concentration being around 500μM) for SC. To examine the role of calcium buffering in the increased excitability of the cells, the authors should perform experiments with high concentrations of intracellular BAPTA (10mM) while maintaining resting calcium levels and evaluate if change in cell excitability is still present.

2-Previous studies demonstrated that the major anions of the intracellular solution (here: gluconate) could impact the function of certain ion channels. The authors should provide experimental data demonstrating how the intrinsic excitability is affected by different anions (e.g. methanesulfonate).

3-Most of the experiments were performed at room temperature. Is the increase in excitability also observed at elevated temperatures 34-35? This is particularly relevant given that temperature is likely to modify the functional properties of ion channels and potential sensitivity to intracellular modulators. If the authors do not have experimental data obtained at elevated temperatures, they should at least discuss its potential effects in the Discussion.

Minor points:

Line 117 “Using a combination of patch-clamp electrophysiology from acutely-isolated tissue...”

Would be clearer as “electrophysiology from brain-slice recording...”. The wording made me think that the authors were going to use a preparation of acutely-dissociated neurons.

Line 130 “ All experiments have been approved by the local authorities...” This is very vague and needs to be more specific.

Line 233 “The model is six-dimensional, consisting of 5 ionic currents, including ᵃC;ᵄ1;ᵄE; , ᵃC;ᵃE; , ᵃC;ᵃ4;, T-type Ca2+ current (ᵃC;ᵄ7; ) and nonspecific leak current (ᵃC;ᵃF; ).” How is the model 6 dimensional if it has 5 ionic currents?

Line 295 “to keep the formalism of the model in Molineux et al. (2005) and Anderson et al. (2010) unaltered, we chose ᵄ9;1/2 values for the activation and inactivation of ᵃC;ᵃ4; in the dynamic model similar to those obtained here...” The wording is ambiguous. I suppose the authors mean “similar to those given there” -meaning in the Molineux and Anderson papers. “Obtained here” sounds like they mean ones they obtained, here in the present work, but this does not make sense in the context of the sentence.

Line 811, Fig leg 1. “Figure 1: Stellate and granule cells, but not Purkinje cells, exhibit excitability increases over patch-clamp recording”. “During whole-cell recording” would be more precise.

What is the free resting calcium concentration in the intracellular solution used for current clamp recordings? This should be mentioned in the methods together with the way it was calculated.
